# Overexpression of thioredoxin in islets transduced by a lentiviral vector prolongs graft survival in autoimmune diabetic NOD mice

**DOI:** 10.1186/1423-0127-16-71

**Published:** 2009-08-12

**Authors:** Feng-Cheng Chou, Huey-Kang Sytwu

**Affiliations:** 1 Graduate Institute of Life Sciences, National Defense Medical Center, Taipei, Taiwan, Republic of China; 2 Graduate Institute of Medical Sciences, National Defense Medical Center, Taipei, Taiwan, Republic of China

## Abstract

**Abstract:**

Pancreatic islet transplantation is considered an appropriate treatment to achieve insulin independence in type I diabetic patients. However, islet isolation and transplantation-induced oxidative stress and autoimmune-mediated destruction are still the major obstacles to the long-term survival of graft islets in this potential therapy. To protect islet grafts from inflammatory damage and prolong their survival, we transduced islets with an antioxidative gene *thioredoxin (TRX) *using a lentiviral vector before transplantation. We hypothesized that the overexpression of TRX in islets would prolong islet graft survival when transplanted into diabetic non-obese diabetic (NOD) mice.

**Methods:**

Islets were isolated from NOD mice and transduced with lentivirus carrying *TRX *(Lt-*TRX*) or enhanced green fluorescence protein (Lt-e*GFP*), respectively. Transduced islets were transplanted under the left kidney capsule of female diabetic NOD mice, and blood glucose concentration was monitored daily after transplantation. The histology of the islet graft was assessed at the end of the study. The protective effect of TRX on islets was investigated.

**Results:**

The lentiviral vector effectively transduced islets without altering the glucose-stimulating insulin-secretory function of islets. Overexpression of TRX in islets reduced hydrogen peroxide-induced cytotoxicity *in vitro*. After transplantation into diabetic NOD mice, euglycemia was maintained for significantly longer in Lt-TRX-transduced islets than in Lt-eGFP-transduced islets; the mean graft survival was 18 vs. 6.5 days (n = 9 and 10, respectively, p < 0.05).

**Conclusion:**

We successfully transduced the *TRX *gene into islets and demonstrated that these genetically modified grafts are resistant to inflammatory insult and survived longer in diabetic recipients. Our results further support the concept that the reactive oxygen species (ROS) scavenger and antiapoptotic functions of TRX are critical to islet survival after transplantation.

## Background

Autoimmune diabetes is an inflammatory disease that causes the loss of insulin-secreting β-cells and hyperglycemia. Islet transplantation can provide near perfect, moment-by-moment control of the homeostasis of blood glucose concentration and is much more effective than insulin injection, which cannot prevent nephropathy, retinopathy, vascular, and heart disease. However, inflammation, allorejection, and recurrent autoimmune damage can contribute to early graft loss and are major obstacles to successful islet transplantation [1]. The process of islet isolation also triggers a cascade of stressful events in the islets involving the induction of apoptosis or necrosis and production of proinflammatory molecules that negatively influence islet viability and function. Proinflammatory cytokines such as IL-1β and TNF-α produced by islet-resident macrophages are toxic to islets and can induce ROS formation in islet cells [2,3]. Inflammatory cytokines and free oxygen radicals released in situ can cause apoptosis and loss of islets after implantation, and eventually graft failure [4,5].

Previous studies have demonstrated that by using different strategies to protect islet from those detrimental immune response result in greatly improving graft function and prolonging graft survival. These strategies include modulating immune response by CTLA-4-Ig or TGF-β [6,7], inflammatory blockade by chemicals [2,8], overexpressing antiapoptotic gene Bcl-2 [9], and reducing oxidative stress by overexpression of antioxidative genes [10]. However, different animal models used made it difficult to compare with each work.

Because islets produce very low antioxidative enzymes and are very sensitive to oxidative stress, clearance of ROS is crucial in the viability of islet graft. The concept of antioxidative treatment in islets has been proven in many models, including the transgenic mouse model with specific expression of antioxidative genes in islets or islet grafts transduced by viral vector carrying those genes. Transgenic expression of antioxidant genes in islets, such as glutathione peroxidase, different isoforms of superoxide dismutase, metallothionein, or catalase, significantly increases the islet viability and reduces ROS formation after challenge with free radical donor or hypoxia exposure *in vitro *[11-13]. The role of ROS scavengers in islet transplantation has also been investigated in these transgenic mouse models using syngeneic or allogeneic islet transplantation. However, only islets from metallothionein transgenic mice showed prolonged islet graft survival in an allogeneic transplantation model [13]. In summary, most results regarding that overexpression of antioxidative genes in islets protects them from oxidative injury were obtained from in vitro experiments, the *in vivo *function and survival of these genetically-modified islets in diabetic recipients was not clear.

Thioredoxin (TRX) is a small, ubiquitously expressed protein in the cell. TRX has many biological functions including the regulation of the cellular reduction-oxidation balance, promotion of cell growth, inhibition of apoptosis, and regulation of gene expression [14-16]. Previous studies demonstrated that administration of recombinant TRX protein reduces brain damage induced by focal cerebral ischemia in mice [17]. In other studies, transgenic overexpression of TRX by the β-actin promoter protected from the disease or reduced the disease severity in different disease models such as acute hepatitis [18] and ischemic brain injury [19] which are caused mainly by oxidative stress, indicating that TRX has strong cytoprotective properties. TRX with antioxidative and antiapoptotic functions has been demonstrated to prevent β cells from autoimmune destruction in a β cell-specific TRX transgenic NOD model, strongly suggesting that oxidative stress plays an essential role in the destruction of β cells by infiltrating inflammatory cells in pancreas [20]. These results suggested that TRX is a better candidate gene than other antioxidative genes which may have therapeutic application in prevention of islet grafts from inflammatory insults. To improve islet grafts viability and maintain euglycemia after islet transplantation in autoimmune diabetes, we used a lentiviral vector delivery system to carry the *TRX *gene into islets before transplantation. In the present study, islet cells overexpressing TRX were more resistant to hydrogen peroxide (H_2_O_2_)-induced cell toxicity and had prolonged islet survival after transplant into diabetic recipients. Our results show that controlling the inflammation-mediated and ROS-mediated islet graft damage is critical for successful islet transplantation.

## Methods

### Animals

NOD/Sytwu (K^d^, D^b^, L^d^, I-A^g7^) mice were purchased originally from Jackson Laboratory (Bar Harbor, ME, USA) and were subsequently bred and maintained under specific pathogen-free conditions at the Animal Center of the National Defense Medical Center (Taipei, Taiwan), which was accredited by AAALAC. Male mice aged 5–8 weeks were used as islet donor and female mice with blood glucose concentration 300–500 mg/dl were selected as recipients.

### Construction of the plasmid and generation of the lentivirus

Human TRX cDNA was cloned from A549 (human lung carcinoma) cells using the primer pair 5'-ggaattcTTTCCATCGGTCCTTACAGC-3' and 5'-gaattcGCAGATGGCAACTGGGTTTA-3', and inserted into a pTYEF-transducing SIN vector. VSV-G-pseudotyped recombinant HIV-based virus was produced by three-plasmid cotransfection of TE671 cells with the packaging helper construct, pHP, the envelope expression construct, pHEF-VSV-G, and the transducing self-inactivating vector carrying the target gene under control of the elongation factor-1α promoter. The viruses were concentrated by ultracentrifugation and titered by transduction of confluent TE671 cells as described before [21].

### Islet isolation and viral transduction

Islets were purified from 6-week-old male NOD mice using the collagenase-digesting method as described previously [22]. Briefly, the common bile duct was clamped at its entrance to the duodenum, and 2.5 ml of cold Hank's balanced salt solution containing 1.5 mg/ml of collagenase XI (Sigma-Aldrich, St Louis, MO, USA) was injected into the common bile duct. The islets were incubated in a 37°C water bath for 20 min and then separated by a density gradient using Histopaque 1077–1 (Sigma-Aldrich). Finally, islets with a diameter between 75 μm and 250 μm were handpicked under a dissecting microscope and confirmed by dithizone (Sigma-Aldrich) staining. Purified islets were suspended in 0.5 ml of culture medium containing 8 μg/ml polybrene (Sigma-Aldrich) and infected with lentivirus at a multiplicity of infection (MOI) of 10. Islet is a 3-dimensional architecture which is composed by α, β, δ, ε and PP cells and contains on average 1000 cells. The MOI was calculated according to the assumption that islets contain on average 1000 cells. Islets were incubated at 37°C for 3 h with lentivirus in about 0.5 ml of medium and then cultured in F12K (Invitrogen, Carlsbad, CA, USA) medium supplemented with 10% fetal bovine serum, 1% penicillin-streptomycin (10,000 units/ml) (Invitrogen), and 1% L-glutamine (2 mmol/l) (Invitrogen) at 37°C in 5% CO_2 _before use for transplantation or *in vitro *analysis. When using the replication-defective lentiviral vectors to infect islets, only part of cells surrounded of the islet could be efficiently infected and the transduction efficiency is around 5% of total islet cells [23].

### In vitro studies of islet cell function

After incubation with lentivirus for 24 h, islets were washed with RPMI-1640, and a glucose-stimulated insulin-secretion test was performed using Millicell^® ^Cell Culture Inserts (Corning Inc., Corning, NY, USA). Twenty-five islets were cultured in the Millicell inserts, washed with glucose-free RPMI-1640, and then preincubated in RPMI-1640 containing 2.8 mM glucose for 30 min. Islets were stimulated first with RPMI-1640 containing 2.8 mM glucose for 1 h and then moved to a second well containing 16.7 mM glucose for an additional 1 h. Insulin released into the culture medium was measured by ELISA (Mercodia, Uppsala, Sweden).

### MTT cell viability assay

Forty-eight hours after virus transduction, islets were challenged with H_2_O_2 _at different concentrations (50, 75, and 100 μM) for 18 h. Islet cell viability was measured by the MTT assay (Sigma-Aldrich) as described previously [24].

### RNA extraction and quantitative PCR analysis

Total RNA was extracted using TRIzol reagent (Invitrogen) and reverse transcribed to cDNA using SuperScript™ III Reverse transcriptase kit (Invitrogen). PCR for TRX was run at 35 cycles (45 s of denaturation at 94°C, 45 s of annealing at 55°C, and 45 s of extension at 72°C), and the PCR products were separated on a 1.2% agarose gel. The cDNA was used as a template in the subsequent PCR analyses. Transcript levels were determined by real-time PCR using Bio-Rad iCycler and iQ SYBR Green Supermix (Bio-Rad, Hercules, CA, USA). PCR primers for TRX were forward 5'-GGAAT TCTTTCCATCGGTCCTTACAGC-3' and reverse 5'-GGAATTCGCAGATGGCAACTGGGTTTA-3'. Primers for real-time PCR were list below: forward 5'-GCCACCAAGGAGGTACACAT-3' and reverse 5'-GCTTGTTGCGCTCTATCTCC-3' for HO-1, forward 5'-ATCCTTGGAGCCAGTCAAGA-3' and reverse 5'-ATGATGCCGGAAACAAGAAG-3' for c-fos, forward 5'-TCCCCTATCGACATGGAGTC-3' and reverse 5'-TTTTGCGCTTTCAAGGTTTT-3' for c-jun, forward 5'-ACGGTCTGATCCGCAAATAC-3' and reverse 5'-AGCATGATCGGTTCCACTTG-3' for Rps29 (housekeeping gene).

### Transient transfection of reporter gene system

Lt-eGFP- or Lt-TRX-transduced NIT-1 cells (1 × 10^5^/well) were seeded and maintained in 24-well plate for 24 hours in F-12K medium and were then transfected with pCMV-luciferase (Promega, Milan, Italy) and pAP1-SEAP (Clontech, San Jose, CA) at a ratio of 1:50 for 4 hours using the Lipofectamine 2000 (Invitrogen, Carlsbad, CA, USA) following the manufacturer's recommendations. Transfected cells were maintained for 20 hours in the complete medium and then cultured in serum free F-12K medium for an additional 24 hours. The SEAP activity was determined in culture supernatants, and luciferase activity was measured in cell lysates to normalize the transfection efficiency.

### Immunoblot analysis of human TRX expression

Total protein from cellular lysate was prepared and size-fractionated by 15% sodium dodecyl sulfate-polyacrylamide gel electrophoresis and electroblotted to onto a nitrocellulose membrane. The human TRX protein was detected using the goat anti-human TRX antibody (R&D systems, Minneapolis, MN).

### Islet transplantation

Twenty-four hours after virus transduction, marginal islets were collected and washed, and a total of around 700 islets were implanted into the left renal capsule of newly diabetic NOD female mice whose blood glucose concentration was 300–500 mg/dl. Blood glucose concentration was monitored daily after islet transplantation. Loss of graft function was defined as a blood glucose concentration > 300 mg/dl on two consecutive days.

### Histological analysis of the grafts

Graft-bearing kidneys were removed on day 7 after transplantation and embedded in OCT for frozen sectioning. The sections were stained with hematoxylin and eosin (Sigma-Aldrich). For immunohistochemical analysis, tissue sections were stained with either an insulin (Abcam), TRX (R&D systems, Minneapolis, MN) or HO-1 (Stressgen, Ann Arbor, MI) primary antibody and followed by an HRP-conjugated secondary antibody. Chromogen substrate 3-3' diaminobenzidine (DAKO, Carpinteria, CA) was added for enzymatic stain development which resulted in a brown colored precipitate at the antigen sites. Mayer's hematoxylin was applied as a counterstain.

### Statistical analysis

Insulin secretion and islet viability data were analyzed using Student's *t *test and islet graft survival was analyzed using the Kaplan-Meier method. A p value < 0.05 was defined as significant.

## Results

### TRX transgene expression in lentiviral vector-transduced islets

**Figure 1:**
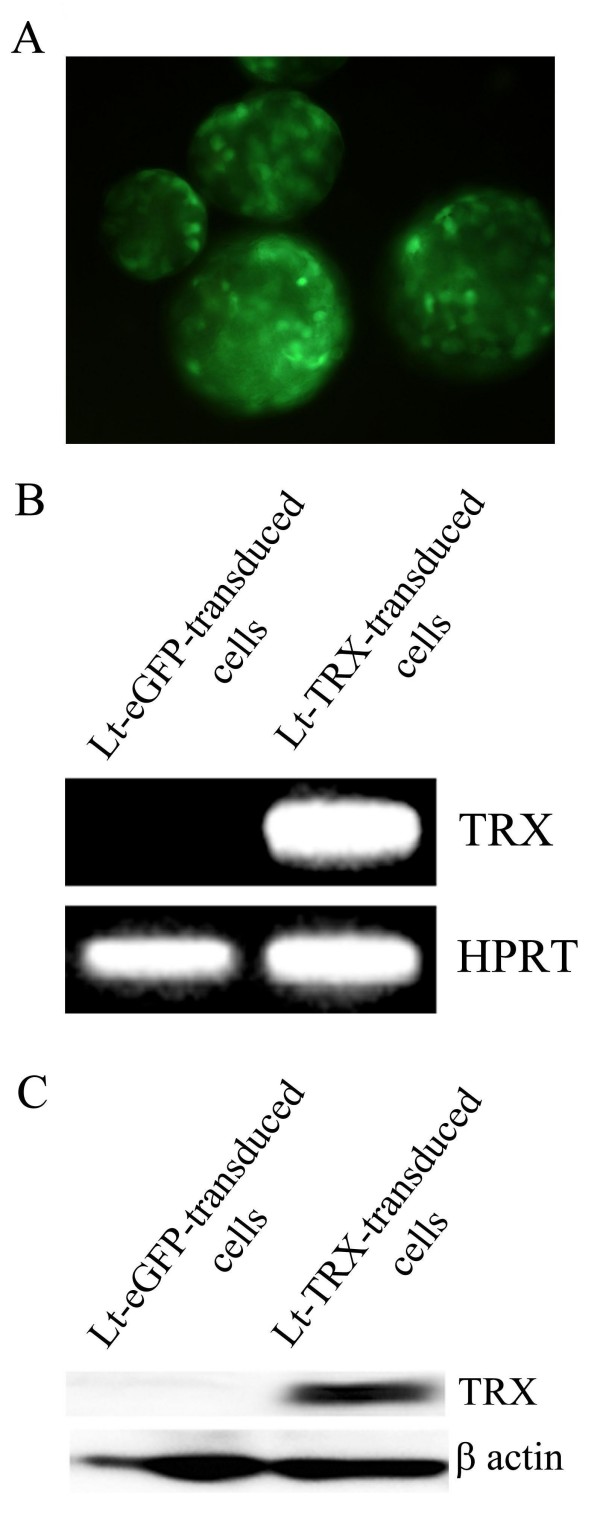
**Lentiviral vector-transduced islets**. (A) Fluorescence microscope image displays the eGFP protein expression in Lt-eGFP-transduced islets. Original magnification × 200. (B) RT-PCR analysis demonstrates TRX mRNA expression in Lt-TRX-transduced islets. MOI = 10.

The lentiviral vector is an excellent tool for experimental gene transfer because it infects both dividing and nondividing cells such as pancreatic islets. The transgene carried by this vector can be stably expressed over the long term in host cells after virus transduction [25]. To investigate the transduction efficiency of the lentivirus in the freshly isolated islets from NOD mice, we transduced islets with lentivirus at various MOIs and checked the mRNA and protein levels of the transgene expression after virus transduction. Islets were isolated from 6-week-old male NOD mice and morphologically intact islets were selected for the experiment. Islet cells were transduced with the lentivirus carrying eGFP or TRX. Transgene expression was confirmed by fluorescent microscopic imaging for eGFP protein expression (Fig. 1a) and RT-PCR analysis for human TRX mRNA expression (Fig. 1b) in lentivirus-transduced islets. The TRX protein expression in Lt-TRX-transduced islets was confirmed by an immunoblot analysis (Fig. 1c). At an MOI of 10, transgenes were expressed successfully in the islets, and we used an MOI of 10 to transduce islets in further experiments. Our data demonstrate that the lentivirus delivered the target genes into islets effectively and that it has potential as a tool for genetic manipulation in these almost nondividing islets.

### Insulin-secreting function of islets after lentivirus transduction

**Figure 2:**
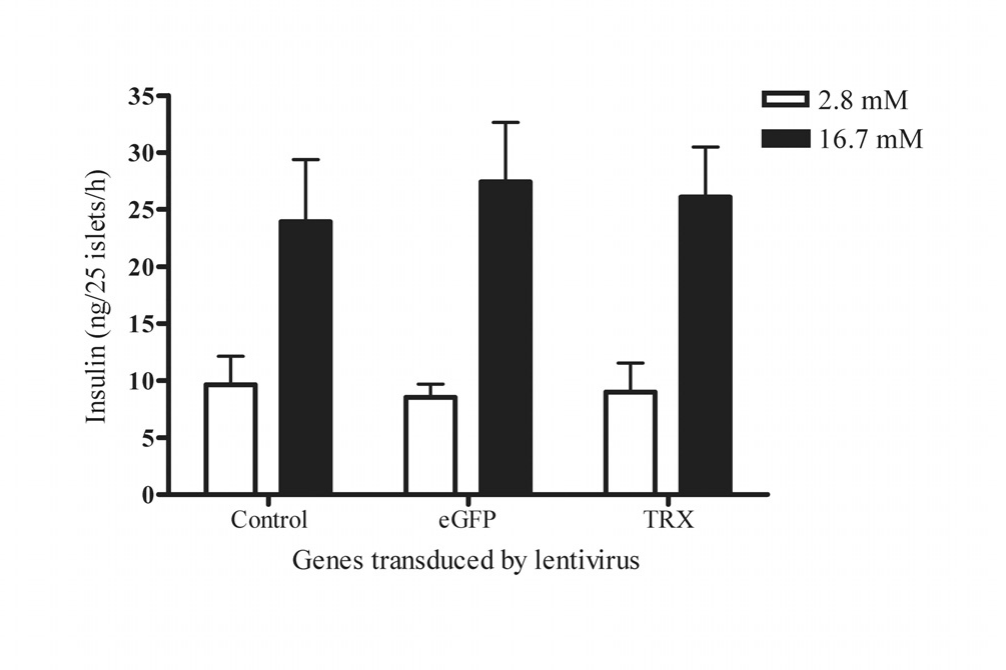
**Insulin-secreting function of islets**. Freshly isolated islets were transduced with Lt-eGFP or Lt-TRX. After 24 h culture, the glucose-stimulated insulin-secretion test was performed. The concentration of insulin released into the medium was measured by ELISA. The concentration of insulin release is expressed as mean ± SD of three independent experiments.

The insulin-secreting function of islets is tightly regulated in response to environmental glucose concentration, and this control is important for glucose homeostasis. To evaluate whether the insulin-secreting function was affected in lentivirus-transduced islets, a glucose-induced insulin-secretion assay was performed. One day after virus transduction, islets of similar size and with intact morphology were selected for the glucose-induced insulin-secretion assay. The concentration of insulin released into the medium was measured by ELISA. The concentration of insulin released into the medium did not differ between control islets and lentivirus-transduced islets (Fig. 2). Our data demonstrate that overexpression of eGFP or TRX in islets did not alter the glucose-stimulated insulin-secreting function of islets, suggesting that *in vitro *manipulation of islets by lentivirus did not affect their health or physiological function

### H_2_O_2_-induced cytotoxicity in Lt-eGFP- or Lt-TRX-transduced islets

**Figure 3:**
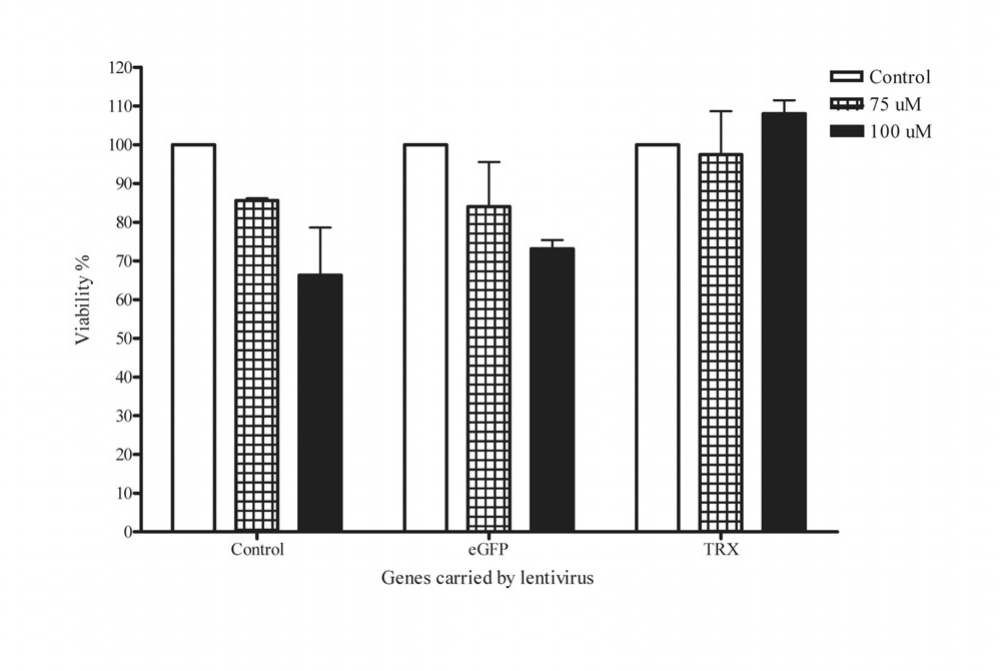
**Overexpression of TRX reduces H_2_O_2_-induced cytotoxicity**. Freshly isolated islets were transduced with Lt-eGFP or Lt-TRX. After 48 h culture, islets were treated with H_2_O_2 _at different concentrations for 18 h. Islet cell viability was measured by the MTT assay. The viability results are expressed as mean ± SD of three independent experiments.

TRX regulates the cellular redox status through its two cysteine residues and counteracts the toxic effect of ROS. H_2_O_2 _is toxic to cells at low concentrations, and this toxicity can be neutralized by TRX. To evaluate the protective effect of transgenic TRX on the islet damage mediated by oxidative stress, we used H_2_O_2 _as a ROS donor to induce islet cell death and investigated the protective effect of TRX on islets under H_2_O_2 _stimulation. Lt-eGFP- and Lt-TRX-transduced islets were incubated with various concentrations of H_2_O_2 _for 18 h, and the MTT assay was performed to evaluate the islet cell viability. Islet cells overexpressing TRX were more resistant to H_2_O_2_-induced cytotoxicity at a concentration of 100 μM (Fig. 3). These data indicate that overexpression of TRX in islets by the lentiviral vector did not affect the insulin-secreting function of islets and further protected the islets from exogenous stress stimulation, suggesting that TRX is a potential candidate for genetic manipulation of islet grafts in transplantation.

### Survival of TRX-transduced islet grafts after transplantation

**Figure 4:**
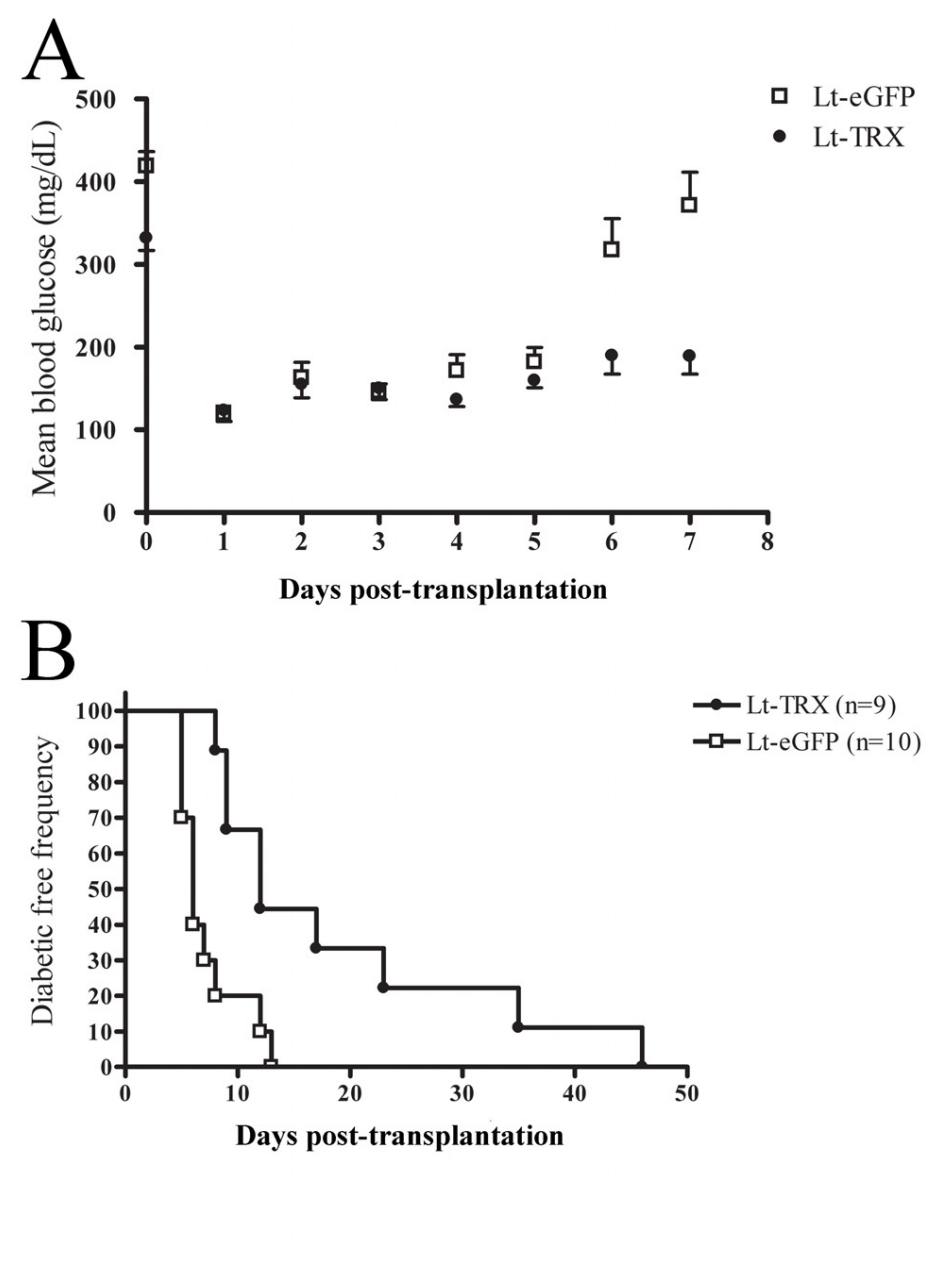
**Function of marginal islets and diabetic-free frequency of diabetic recipients**. (A) Mean blood glucose concentration in mice that received Lt-eGFP- or Lt-TRX-transduced islets. Data are expressed as the mean ± SD from 10 mice in each group. (B) Lt-TRX-transduced islets maintained euglycemia for significantly longer than in the Lt-eGFP-transduced group. Blood glucose concentration was monitored daily after islet implantation. Loss of graft function was defined as a blood glucose concentration > 300 mg/dl on two consecutive days.

**Figure 5:**
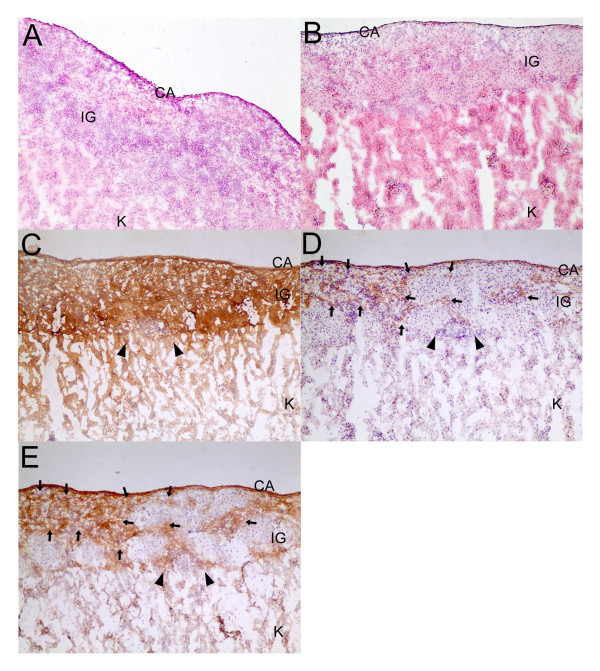
**Histology of islet grafts (IG) under the capsule (CA) of the kidney (K)**. Lt-eGFP- and Lt-TRX-transduced islets were transplanted into diabetic NOD mice in the same operation. IG-implanted kidneys were removed on day 7 when the Lt-TRX-transduced IG was still functional and maintained euglycemia, but the Lt-eGFP-transduced IG had failed. (A) Severe leukocytic penetration was observed in Lt-eGFP-transduced IG. (B) Lt-TRX-transduced IG had less severe leukocytic infiltration and islet cells were still visible. Serial sections of Lt-TRX transduced islet grafts were immunohistochemically stained with insulin (C), TRX (D) and HO-1 (E). Arrows in (D) and (E) point out examples of the TRX and HO-1 staining respectively and arrow heads point out the infiltrated lymphocytes. Original magnification × 100.

Oxidative stress induced by inflammation and islet isolation and transplantation are major obstacles to islet replacement therapy because they may cause primary non-function of islets and early graft loss. Considering the advantages of the antioxidative and antiapoptotic functions of TRX, we hypothesized that overexpression of this gene would protect islets from the stress induced by the transplantation procedure and subsequent autoimmune attack. Freshly isolated islets were transduced with Lt-TRX or Lt-eGFP and cultured for 1 day. Around 700 marginal islets were transplanted under the left kidney capsule of newly diabetic female NOD mice. Islet grafts with TRX transduction showed better glycemic control (Fig. 4a) and prolonged islet graft survival in diabetic recipients (mean graft survival days, 18 vs. 6.5, n = 9 and 10, respectively, p < 0.05) (Fig. 4b). We also performed nephrectomy of the left kidney implanted with grafted islets to confirm further the glucose-regulatory function of the Lt-TRX-transduced islet grafts. The NOD recipients with normal glycemia became hyperglycemic after the islet-implanted kidney was removed, indicating that the islet grafts were still functional and maintained the normal glycemia in transplanted recipients (data not shown). Histological analysis of the graft-bearing kidneys revealed that although Lt-TRX-transduced islets showed much less leukocytic infiltration than the Lt-eGFP-transduced islets, they were not completely free from lymphocyte infiltration (Fig. 5a and 5b). Immunohistochemical staining showed that Lt-TRX transduced islet grafts were insulin-producing (Fig. 5c) and TRX-expressing (Fig. 5d), suggesting that these islets with transgene expression were still functional. Since heme oxygenase-1 (HO-1) mediates a strong cytoprotection in cells under a variety of stresses, the induction of HO-1 in TRX-transduced islets may have a synergistic effect with TRX to protect them from apoptosis and improve islet survival and function after transplantation. Results from immunohistochemical staining revealed that the distribution of HO-1-expressing cells was quite close to that of the TRX-expressing cells (Fig. 5e), consistent with the previous reports that TRX facilitates the induction of HO-1 [26]. These results indicate that overexpression of TRX in islet grafts protected them from inflammation-induced stress and prolonged graft survival after transplantation, although it could not inhibit leukocytic infiltration completely and prevent loss of the grafts.

### Cellular responses to the TRX overexpression

**Figure 6:**
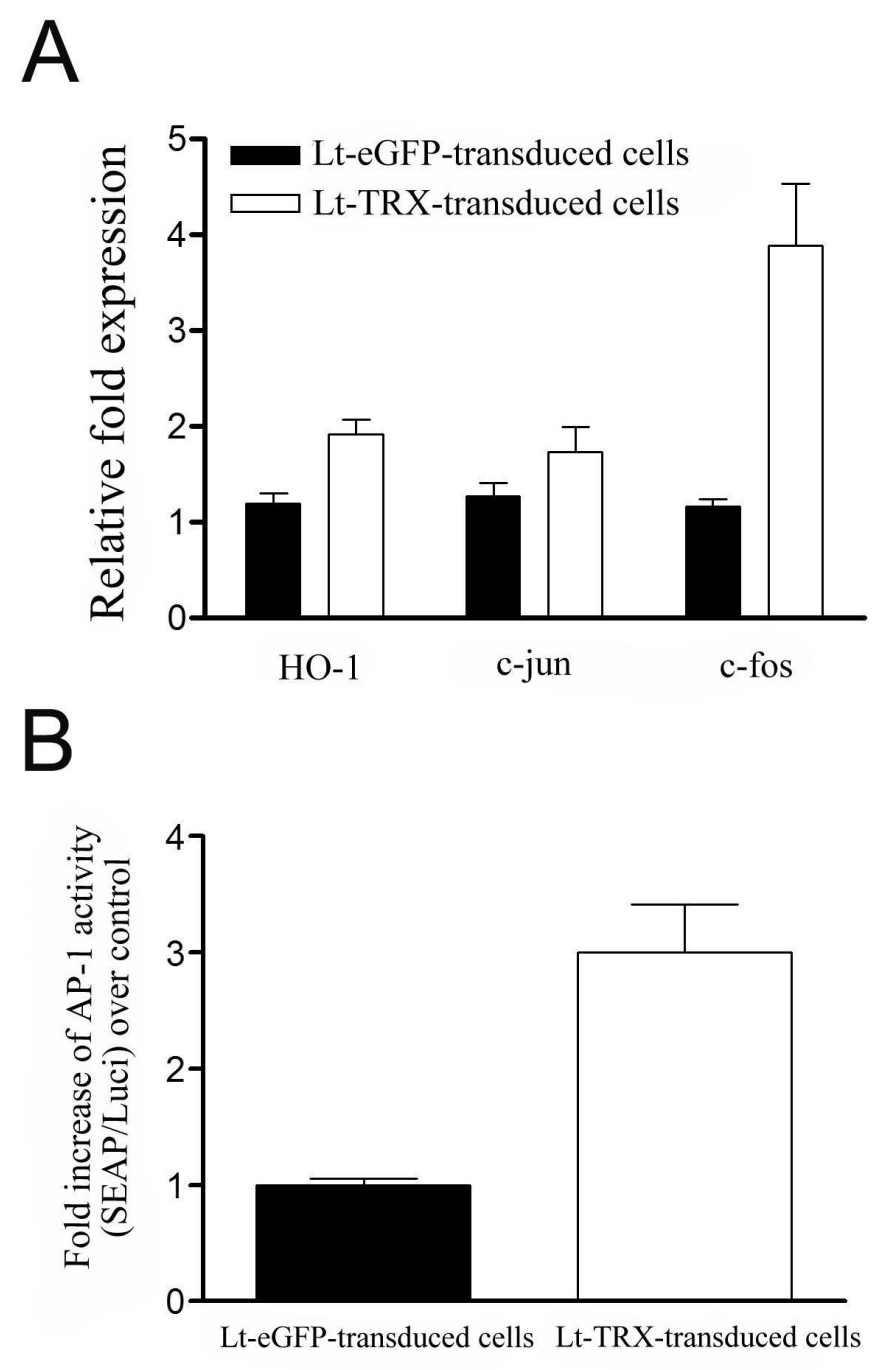
**Cellular responses to TRX overexpressing**. (A) Levels of mRNA of HO-1, c-jun and c-fos genes in Lt-eGFP- or Lt-TRX-transduced cells. The mRNA was measured by real-time PCR. Results are shown as a ratio of indicated gene to Rps29 (housekeeping gene). Results are the mean ± S.E.M. of triplicates from three independent experiments. (B) Lt-eGFP- or Lt-TRX-transduced NIT-1 cells were transiently transfected with pCMV-luciferase and pAP1-SEAP plasmids. SEAP activity in culture supernatants was determined and normalized for luciferase activity in the cell lysates. Data are shown as means ± S.E.M. of triplicates from three independent experiments.

Previous reports have demonstrated that TRX selectively activates a number transcription factors and facilitates the induction of cytoprotective genes [14], suggesting that TRX-mediated islet graft protection may not only acts through free radical scavenger. To investigate the protective mechanisms mediated by TRX in islet grafts, we further analyzed the expression of genes such as c-fos, c-jun, and HO-1 that were regulated by TRX. We also analyzed AP-1 activity in β cell-derived NIT-1 cells after Lt-TRX virus transduction. Quantitative PCR analysis reveals that expression of HO-1 and c-fos was up-regulated after Lt-TRX transduction (Fig. 6a).

To further determine the AP-1 activity in Lt-TRX-transduced cells, we transfected cells with a SEAP reporter plasmid which containing AP-1 binding sites on the upstream promoter region. The AP-1 activity was around 3-fold increase in Lt-TRX-transduced cells than in Lt-eGFP-transduced cells, suggesting that TRX could regulate gene transcription through the up-regulation of AP-1 activity (Fig. 6b). These results are consistent with previous reports that TRX regulates cellular redox status and promotes cell survival through inducing cell protective genes expression.

## Discussion

Clinical trials of islet transplantation are showing remarkable success since the Edmonton protocol [27] was developed, and this glucocorticoid-free immunosuppressive protocol was replicated successfully [28]. However, inadequate islet donors and recurrent autoimmunity are major obstacles in the treatment of type I diabetes. The transplantation procedure such as collagenase-based islet isolation triggers proinflammatory cytokine and chemokine production by islets, which contributes to early graft loss or primary loss of function [3,29]. In this study, we overexpressed TRX in islet grafts to determine whether this would protect them from oxidative stress-induced cell damage. We found that TRX did not alter the glucose-induced insulin-secreting function of the islets but protected them from ROS challenge. In the islet transplantation experiment, TRX-overexpressed islets showed better glycemic control and longer graft survival, indicating that TRX has a strong cytoprotective effect on islets.

Many approaches have been shown to have protective effects in islets, including the regulation of the immune response by overexpressing anti-inflammatory cytokines or cytokine inhibitors [6,30,31]; and by reducing cellular stress by overexpression of antiapoptotic and antioxidative genes in islets [9,10,12]. These approaches might protect islets from apoptosis or promote islet function *in vitro *or *in vivo*, although the choice of target genes and the methods to overexpress the target genes can affect the results significantly. In general, therapeutic targets that have a paracrine action have a more marked biological effect than do genes that target intracellular molecules. In addition, the selected vectors for carrying the target genes to the islets should have low immunogenicity, and long-term and stable gene expression in islets is required. In this work, we used a lentivirus to carry the TRX gene into the islets, which do not divide or have a low rate of division. This approach has some advantages. First, lentivirus can infect both dividing and nondividing or quiescent cells efficiently and provide for the stable integration into the host cell genome. Second, this method does not elicit an immune response *in vivo*. Third, TRX is a strong ROS scavenger in the cytoplasm and can clear H_2_O_2 _directly and repair the oxidized proteins.

TRX also regulates the expression or activity of other proteins. In some circumstances, TRX translocates to the nucleus to regulate transcription factor activity [32] or can be secreted and act as a growth factor [33]. Under stimulation by inflammatory mediators, TRX regulates AP-1 activity and enhances heme oxygenase-1 (HO-1) expression [26]. The AP-1 activity was around 3-fold increase in Lt-TRX-transduced cells than in Lt-GFP-transduced cells, suggesting that TRX could regulate gene transcription through the up-regulation of AP-1 activity in our model. HO-1 expression was observed in Lt-TRX-transduced islets and induction of HO-1 in islet grafts have been demonstrated to protect them from apoptosis and improve their function after transplantation [34]. Thus, TRX and HO-1 may play coordinated roles in protecting cells from inflammatory stress. The reduced form of TRX also binds to a variety of cellular proteins. Apoptosis signal-regulating kinase 1 (ASK1) is one TRX-binding protein that mediates stress- and cytokine-mediated apoptosis [35]. TRX binds to the N-terminus of ASK1, and activation of ASK1 requires dissociation of the TRX [16,36]. Taken together, the antioxidative and antiapoptotic functions of TRX may contribute to protecting islets from inflammation-induced cell injury.

## Conclusion

It is unlikely that any single biological agent will be sufficient to stop such complicated autoimmune processes such as cytokine imbalance, free radical formation, and cellular apoptosis [37]. Using newly diabetic NOD mice that had developed strong autoimmunity to pancreatic islets as the recipients, we showed that TRX can prolong graft survival significantly. This effect may reflect the multiple biological functions of TRX, which has both antioxidative and antiapoptotic activities. These are important functions for preventing islets from immune attack, and this idea has been demonstrated in the transgenic model [20]. However, despite its beneficial effects, TRX could not inhibit the leukocytic infiltration into the islet grafts completely, and future studies using combinations of immunoregulatory genes may help prolong graft survival and maintain long-term glucose homeostasis in diabetic recipients.

## Competing interests

The authors declare that they have no competing interests.

## Authors' contributions

FCC ried out the molecular genetic studies, participated in the sequence alignment and drafted the manuscript, participated in the design of the study and performed the statistical analysis, conceived of the study, and participated in its design and coordination and helped to draft the manuscript. HKS carried out the molecular genetic studies, participated in the sequence alignment and drafted the manuscript, participated in the design of the study and performed the statistical analysis, conceived of the study, and participated in its design and coordination and helped to draft the manuscript
